# Ethnopharmacology—A Bibliometric Analysis of a Field of Research Meandering Between Medicine and Food Science?

**DOI:** 10.3389/fphar.2018.00215

**Published:** 2018-03-15

**Authors:** Andy Wai Kan Yeung, Michael Heinrich, Atanas G. Atanasov

**Affiliations:** ^1^Oral and Maxillofacial Radiology, Applied Oral Sciences, Faculty of Dentistry, The University of Hong Kong, Hong Kong, Hong Kong; ^2^Research Group “Pharmacognosy and Phytotherapy”, UCL School of Pharmacy, London, United Kingdom; ^3^Institute of Genetics and Animal Breeding, Polish Academy of Sciences, Magdalenka, Poland; ^4^Department of Pharmacognosy, University of Vienna, Vienna, Austria

**Keywords:** bibliometrics, ethnopharmacology, ethnobotany, ethnomedicine, medicinal plant, folk medicine, traditional medicine

## Abstract

**Background:** The research into bioactive natural products of medicinal plants has a long tradition, but ethnopharmacology as a well-defined field of research has a relatively short history, only dating back 50 years.

**Aims:** With the fast development of this field and its global importance especially in the fast developing economies of Asia it is timely to assess the most influential articles (as measured by citations) and to identify important drivers and research trends in this field.

**Methods:** Scopus was searched to identify relevant articles which were assessed by all three authors. The 100 most cited articles were identified and analyzed. Bibliometric software (VOSviewer) was utilized to supplement the analysis and to generate a term map that visualized the citation patterns of the 100 articles containing different terms.

**Results:** Forty-four of the 100 articles are reviews. On average, each of the 100 articles had 632 citations and since publication was cited 43 times annually. The four core journals were *Journal of Ethnopharmacology* (*n* = 17), *Food Chemistry* (*n* = 7), *Life Sciences* (*n* = 5), and *Journal of Agricultural and Food Chemistry* (*n* = 4). Anti-oxidant effects appeared to be a recurring and highly cited topic, whereas the links into drug discovery and neuropharmacology seemed to be less strong. Numerous medicinal plants and functional foods were the foci of research, and the foci shifted when comparing pre-2000 and post-2000 publications (with the later involving a broader spectrum of plants and foods and a wider range of biological effects). Contributions largely came from Asia, and also from the Americas, Africa, and Oceania, besides Europe.

**Conclusion:** We have identified and analyzed the 100 most-cited articles in ethnopharmacology. Within 50 years the field has gained a profile and while conventionally often linked to “traditional knowledge,” drug discovery and some areas of pharmacology, this analysis highlights its emerging importance in the context of disease prevention (food science), but also the development of research driven by the needs and interests of the fast developing economies most notably of Asia.

**Graphical Abstract:**
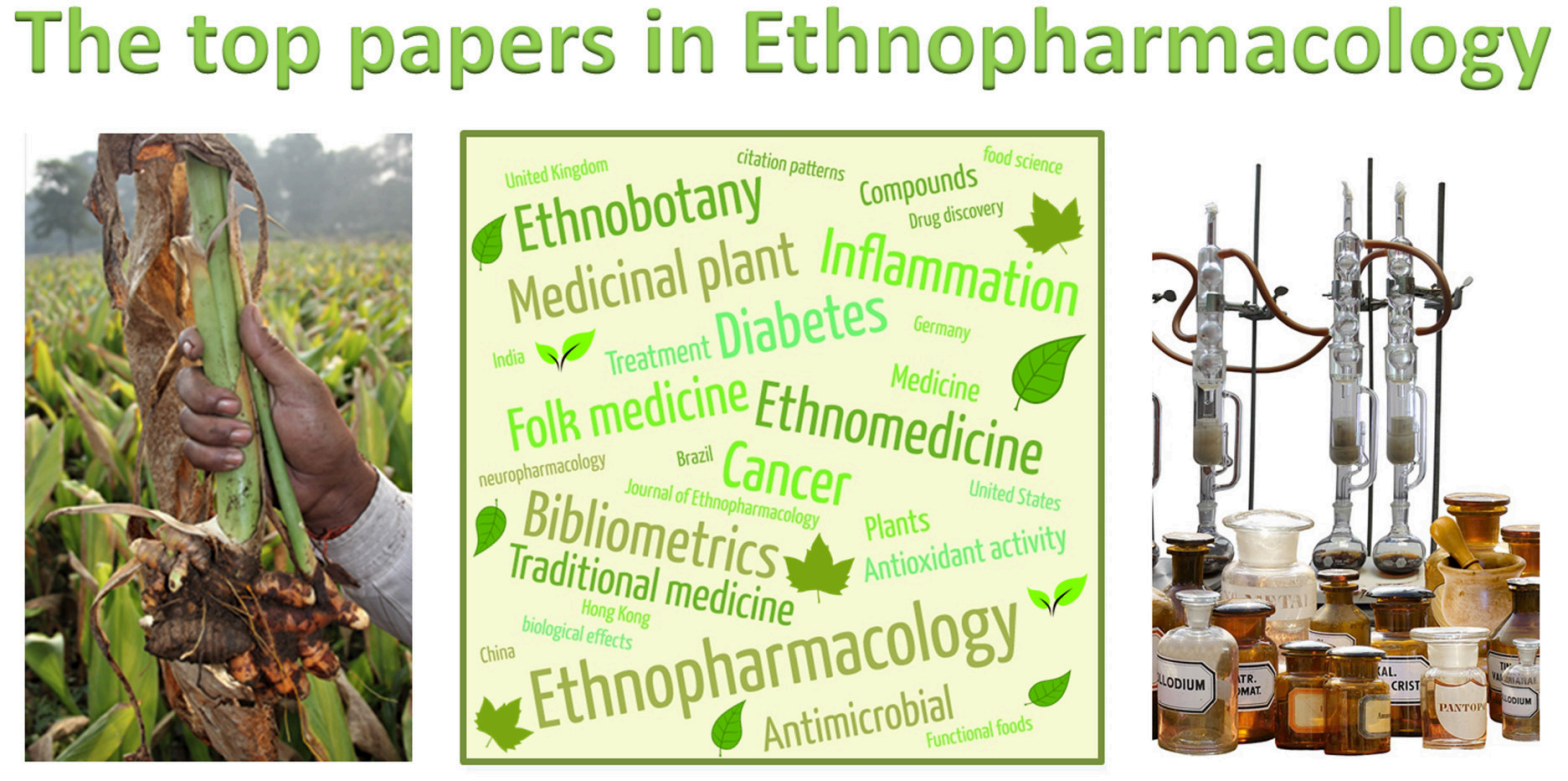
The top papers in ethnopharmacology display a crossover of medical and food science.

## Introduction

Historically, there are abundant and diversified studies reporting on the biological, pharmacological, and medical uses of plants, fungi, and other organisms within a local or traditional context (Heinrich and Jäger, 2015). Many pharmaceutical agents commonly used today originate from medicinal plants, such as aspirin, morphine, pilocarpine, and quinine (Gilani, 2005), and more recently galanthamine, peplin, and Crofelmer (from *Croton lechleri* Müll.Arg.; Heinrich, 2013). Moreover, plants continue to be an important source for modern drug discovery aiming the development of new therapeutics (Atanasov et al., 2015). Instead of being dominated by driving forces from the “Western World,” investigations in ethnopharmacology have next to European (Vogl et al., 2013) also strong global contributions from the Asian, African, and native American traditional medicine (Sheng-Ji, 2001; Steenkamp et al., 2004), such as those originating from China (Rozema et al., 2012), India (Booker et al., 2012), Southeast Asia, for example, Vietnam (Tran et al., 2015), and Mexico (Heinrich, 2000) and several South and Central American countries.

However, ethnopharmacology as a well-defined field of research has a relatively short history, dating back about 50 years only (Heinrich and Jäger, 2015). This offers an opportunity to make an overall assessment and identify important research trends in this field. With such a rich and diversified literature, it is worthwhile to conduct a bibliometric study that examines the top 100 most-cited articles within the research field of ethnopharmacology. Similar studies that reported the 100 most-cited articles have been published for numerous research fields in life science including emergency medicine (Tsai et al., 2006), neuroimaging (Kim et al., 2016), and neuroscience (Yeung et al., 2017a). This allows an understanding of the research trends in the last decades and the ongoing “hot topics.” To the best of the authors' knowledge, no such bibliometric report has been published for ethnopharmacology.

The current study, therefore, aims to identify and analyze the 100 most-cited ethnopharmacology articles. The specific objectives of this analysis were:

1. To understand what key research themes were relevant (as indicated by citations) in ethnopharmacology?
2. To appreciate what we can learn from the highly cited papers in this field in the context of ethnopharmacology's wider and changing relevance within the natural sciences?
3. To identify who and what has driven this research?
4. To assess what we can learn from this for the future development of the field?

## Materials and methods

### Data sources

Scopus was chosen as the source of data since it has a broader coverage on pharmacology journals compared to Web of Science (Gorraiz and Schloegl, 2008). It is a web-based, multidisciplinary database hosted by Elsevier, and it provides bibliometric data of peer-reviewed articles published in the life, social, physical, and health sciences. In November 2017, we searched Scopus to identify articles with the following string: TITLE-ABS-KEY (ethnopharmacology OR ethnopharmacological OR ethnobotany OR ethnobotanical OR ethnomedicine OR ethnomedical OR “medicinal plant” OR “folk medicine” OR “traditional medicine”). This string searched for articles that contain any of these terms or phrases in their title, abstract, or keywords.

The articles were sorted by citation count in descending order. All authors (AWKY, MH, and AGA) screened the titles and abstracts of the articles to exclude those irrelevant to ethnopharmacology in order to finally include the 100 most cited articles. In the current study, we considered as ethnopharmacology papers those focused on traditional medical use or biological and pharmacological activities of plants, fungi, and other organisms used locally or traditionally as a medicine or to improve health. The definition also includes studies aimed to either improving local healthcare through developing products based on such knowledge or studies in the context of drug discovery/development from natural sources which were based on biological resources with a clear and well-defined local or traditional use. We excluded papers which simply contained some of the string keywords but the major focus of the article was not related to ethnopharmacology. These considerations are in line with the definition of ethnopharmacology adopted by the Journal of Ethnopharmacology^1^, Frontiers in Pharmacology (section Ethnopharmacology)^2^ and the ConSEFS advisory group (Heinrich et al., 2018).

No additional restrictions were placed on the type of research model (*in vivo*/*in vitro*), article type (e.g., research article, review, editorial, letter, etc.), or publication language.

### Data extraction

The 100 most-cited articles were evaluated and the following information extracted: (1) publication year; (2) journal title; (3) journal impact factor at the time of publication; (4) SCImago Journal Rank at the time of publication (SJR indicator, which weighs citations by the importance or prestige of the citing journals); (5) total citation count; (6) adjusted citation count (i.e., citation count per year since publication); (7) field-weighted citation impact (FWCI)^3^; (8) authorship; and (9) article type. In addition, the scopes of the involved journals were classified into nine categories: clinical, ethnopharmacology, and medicinal plant research, food research (chemistry etc.), multidisciplinary journals, microbiology and biotechnology, pharmacology and pharmacy, phytochemistry with a link to traditional uses, plant science, and social sciences.

Bradford's law of scattering was applied to the 100 most-cited articles to investigate if a few core journals accounted for publishing one-third of these articles (Vickery, 1948; Yeung et al., 2017c). Based on Bradford's law, three groups of journals should contribute to the publication of the 100 articles when it is equally divided into three portions, whereas the number of journals within the groups should be in the ratio of 1:n:n^2^. For instance, if one journal publishes 33 articles and the next 33 articles are published by four journals, the remaining 33 articles should be published by 16 journals (1:4:16).

### Statistical analysis

We tested if years since publication would influence the number of citations of the articles. It was tested by Pearson's correlation test. Test result with *p* < 0.05 was considered statistically significant.

### Term map

Words in the titles and abstracts of these 100 articles were parsed, analyzed and visualized by VOSviewer (Van Eck and Waltman, 2009), a bibliometric software that visualizes results as bubble maps. Each bubble represented a term or phrase. Irrelevant terms were manually screened and removed (Heersmink et al., 2011; Yeung et al., 2017c). The bubble size indicated its frequency of occurrence. The bubble color indicated the averaged citation counts received by articles containing the term or phrase. A line connects two bubbles if they co-occurred in any of the 100 articles. If two terms co-occurred more frequently, the two bubbles will be in closer proximity. The term map visualizes terms that appeared in at least five of the 100 articles.

## Results and discussion

The 100 most-cited ethnopharmacology articles were mainly original articles (*n* = 51) and reviews (*n* = 44), with a few conference papers (*n* = 3) and short surveys (*n* = 2). Since such a bibliometric assessment is not available for general pharmacology, no direct comparison is possible. However, one can compare the size of the respective fields. A search in Scopus replacing our pre-defined search terms with different ones has revealed that there are 181,207 papers tagged as “pharmacology,” 4,998 papers as “neuropharmacology,” 3,450 papers as “ethnopharmacology,” and 39,848 papers as “traditional medicine/ethnopharmacology.”

The number of citations received by these 100 articles ranged from 353 to 5,253 (mean ± SD: 631.7 ± 560.0, cumulative total citations = 63,166; Table 1). The adjusted citation count (i.e., citation count per year since publication) ranged from 10.8 to 276.5 (mean ± SD: 43.3 ± 31.2, Table 1). Regardless of total citation count or adjusted citation count, Eisenberg et al. (1998) published the top-ranked article, a national survey reporting trends in alternative medicines use in the United States.

**Table 1:** List of 100 most-cited ethnopharmacology articles ranked according to their total citation counts.

**Rank**	**Year**	**Authors and title**	**Journal**	**Impact factor**	**SJR**	**Total citation count**	**Adjusted citation count**	**Field-weighted citation impact**
1	1998	Eisenberg D.M., Davis R.B., Ettner S.L., Appel S., Wilkey S., Van Rompay M., Kessler R.C. Trends in alternative medicine use in the United States, 1990-1997: Results of a follow-up national survey	Journal of the American Medical Association	9.6	NA	5,253	276.5	124.2
2*	1999	Kähkönen MP, Hopia A.I., Vuorela H.J., Rauha J.-P., Pihlaja K., Kujala T.S., Heinonen M. Antioxidant activity of plant extracts containing phenolic compounds	Journal of Agricultural and Food Chemistry	1.5	0.9	2,054	114.1	12.2
3*	1998	Velioglu Y.S., Mazza G., Gao L., Oomah B.D. Antioxidant activity and total phenolics in selected fruits, vegetables, and grain products	Journal of Agricultural and Food Chemistry	1.5	NA	2,034	107.1	7.0
4*	1985	Klayman D.L. Qinghaosu (artemisinin): An antimalarial drug from China	Science	10.9	NA	1,623	50.7	NA
5*	2001	Zheng W., Wang S.Y. Antioxidant activity and phenolic compounds in selected herbs	Journal of Agricultural and Food Chemistry	1.6	1.1	1,345	84.1	9.9
6	1999	Hammer K.A., Carson C.F., Riley T.V. Antimicrobial activity of essential oils and other plant extracts	Journal of Applied Microbiology	1.6	1.1	1,232	68.4	5.1
7	2004	Cai Y., Luo Q., Sun M., Corke H. Antioxidant activity and phenolic compounds of 112 traditional Chinese medicinal plants associated with anticancer	Life Sciences	2.2	0.9	1,224	94.2	11.3
8*	2003	Wasser S. Medicinal mushrooms as a source of antitumor and immunomodulating polysaccharides	Applied Microbiology and Biotechnology	2.1	1.0	1,162	83.0	14.5
9	2002	Grover J.K., Yadav S., Vats V. Medicinal plants of India with anti-diabetic potential	Journal of Ethnopharmacology	1.2	0.9	977	65.1	8.1
10	2004	Miliauskas G., Venskutonis P.R., Van Beek T.A. Screening of radical scavenging activity of some medicinal and aromatic plant extracts	Food Chemistry	1.6	1.1	903	69.5	13.8
11*	2002	Koleva I.I., Van Beek T.A., Linssen J.P.H., De Groot A., Evstatieva L.N. Screening of plant extracts for antioxidant activity: A comparative study on three testing methods	Phytochemical Analysis	1.5	0.8	879	58.6	5.2
12	2005	Cragg G.M., Newman D.J. Plants as a source of anti-cancer agents	Journal of Ethnopharmacology	1.6	0.8	825	68.8	4.9
13	2004	Devasagayam T.P.A., Tilak J.C., Boloor K.K., Sane K.S., Ghaskadbi S.S., Lele R.D. Free radicals and antioxidants in human health: Current status and future prospects	Journal of Association of Physicians of India	0	0.2	819	63.0	5.0
14*	2003	Kalemba D., Kunicka A. Antibacterial and antifungal properties of essential oils	Current Medicinal Chemistry	4.5	1.6	804	57.4	3.0
15*	1996	Haslam E. Natural polyphenols (vegetable tannins) as drugs: possible modes of action	Journal of Natural Products	1.3	NA	797	38.0	6.8
16*	1992	Block E. The organosulfur chemistry of the genus Allium—implications for the organic chemistry of sulfur	Angewandte Chemie International Edition	6.0	NA	770	30.8	NA
17	2005	Edeoga H.O., Okwu D.E., Mbaebie B.O. Phytochemical constituents of some Nigerian medicinal plants	African Journal of Biotechnology	0	0.2	766	63.8	3.5
18	2008	Harvey A.L. Natural products in drug discovery	Drug Discovery Today	6.7	2.0	736	81.8	8.5
19	2001	Fabricant D.S., Farnsworth N.R. The value of plants used in traditional medicine for drug discovery	Environmental Health Perspectives	3.2	1.4	720	45.0	2.8
20	1985	Farnsworth N.R., Akerele O., Bingel A.S., Soejarto D.D., Guo Z. Medicinal plants in therapy	Bulletin of the World Health Organization	1.8	NA	705	22.0	NA
21	2001	Rates S.M.K. Plants as source of drugs	Toxicon	1.6	0.6	689	43.1	1.6
22*	2007	Aggarwal B.B., Sundaram C., Malani N., Ichikawa H. Curcumin: the Indian solid gold.	Advances in Experimental Medicine and Biology	0.7	0.3	669	66.9	9.5
23	2005	Ríos J.L., Recio M.C. Medicinal plants and antimicrobial activity	Journal of Ethnopharmacology	1.6	0.8	665	55.4	3.7
24	2006	Gurib-Fakim A. Medicinal plants: Traditions of yesterday and drugs of tomorrow	Molecular Aspects of Medicine	0	2.2	651	59.2	4.1
25	2005	Balunas M.J., Kinghorn A.D. Drug discovery from medicinal plants	Life Sciences	2.6	0.9	641	53.4	11.7
26	1997	Parmar V.S., Jain S.C., Bisht K.S., Jain R., Taneja P., Jha A., Tyagi O.D., Prasad A.K., Wengel J., Olsen C.E., Boll P.M. Phytochemistry of the genus Piper	Phytochemistry	1.2	NA	620	31.0	3.4
27	2004	Sparg S.G., Light M.E., Van Staden J. Biological activities and distribution of plant saponins	Journal of Ethnopharmacology	1.5	0.8	615	47.3	3.4
28	2004	Amarowicz R., Pegg R.B., Rahimi-Moghaddam P., Barl B., Weil J.A. Free-radical scavenging capacity and antioxidant activity of selected plant species from the Canadian prairies	Food Chemistry	1.6	1.1	595	45.8	7.9
29	2006	Djeridane A., Yousfi M., Nadjemi B., Boutassouna D., Stocker P., Vidal N. Antioxidant activity of some Algerian medicinal plants extracts containing phenolic compounds	Food Chemistry	2.5	1.4	593	53.9	11.2
30	2000	Nascimento G.G.F., Locatelli J., Freitas P.C., Silva G.L. Antibacterial activity of plant extracts and phytochemicals on antibiotic-resistant bacteria	Brazilian Journal of Microbiology	0	0.2	591	34.8	0.6
31	2003	Petersen M., Simmonds M.S.J. Rosmarinic acid	Phytochemistry	1.9	0.9	590	42.1	3.5
32	2006	Cos P., Vlietinck A.J., Berghe D.V., Maes L. Anti-infective potential of natural products: how to develop a stronger *in vitro* 'proof-of-concept'	Journal of Ethnopharmacology	1.7	1.0	589	53.5	4.5
33	2010	Lobo V., Patil A., Phatak A., Chandra N. Free radicals, antioxidants and functional foods: impact on human health	Pharmacognosy Reviews	0	0.2	586	83.7	4.5
34	2007	Lansky E.P., Newman R.A. Punica granatum (pomegranate) and its potential for prevention and treatment of inflammation and cancer	Journal of Ethnopharmacology	2.1	0.9	581	58.1	6.6
35	2001	Ahmad I., Beg A.Z. Antimicrobial and phytochemical studies on 45 Indian medicinal plants against multi-drug resistant human pathogens	Journal of Ethnopharmacology	0.8	0.7	568	35.5	2.6
36	1998	Hande K.R. Etoposide: Four decades of development of a topoisomerase II inhibitor	European Journal of Cancer	2.8	NA	566	29.8	3.7
37	1998	Ahmad I., Mehmood Z., Mohammad F. Screening of some Indian medicinal plants for their antimicrobial properties	Journal of Ethnopharmacology	0.6	NA	564	29.7	1.2
38*	1989	Bailey C.J., Day C. Traditional plant medicines as treatments for diabetes	Diabetes Care	2.4	NA	560	20.0	NA
39	2006	Katalinic V., Milos M., Kulisic T., Jukic M. Screening of 70 medicinal plant extracts for antioxidant capacity and total phenols	Food Chemistry	2.5	1.4	557	50.6	15.5
40*	1999	Wasser S.P., Weis A.L. Therapeutic effects of substances occurring in higher basidiomycetes mushrooms: a modern perspective	Critical Reviews in Immunology	5.8	3.7	541	30.1	2.2
41*	2001	Yildirim A., Mavi A., Kara A.A. Determination of antioxidant and antimicrobial activities of *Rumex crispus* L. extracts	Journal of Agricultural and Food Chemistry	1.6	1.1	540	33.8	4.6
42	2010	Chen S., Yao H., Han J., Liu C., Song J., Shi L., Zhu Y., Ma X., Gao T., Pang X., Luo K., Li Y., Li X., Jia X., Lin Y., Leon C. Validation of the ITS2 region as a novel DNA barcode for identifying medicinal plant species	PLoS ONE	4.5	2.7	530	75.7	24.3
43*	1969	Chihara G., Maeda Y., Hamuro J., Sasaki T., Fukuoka F. inhibition of mouse sarcoma 180 by polysaccharides from Lentinus edodes (Berk.) sing.	Nature	NA	NA	519	10.8	NA
44	2003	Yeh G.Y., Eisenberg D.M., Kaptchuk T.J., Phillips R.S. Systematic review of herbs and dietary supplements for glycemic control in diabetes	Diabetes Care	7.6	3.2	507	36.2	5.8
45	1999	Surh Y.-J. Molecular mechanisms of chemopreventive effects of selected dietary and medicinal phenolic substances	Mutation Research—Fundamental and Molecular Mechanisms of Mutagenesis	0	1.0	507	28.2	9.9
46*	2008	Nassiri Asl M., Hosseinzadeh H. Review of pharmacological effects of *Glycyrrhiza* sp. and its bioactive compounds	Phytotherapy Research	1.8	0.7	505	56.1	6.9
47	2008	Ali B.H., Blunden G., Tanira M.O., Nemmar A. Some phytochemical, pharmacological and toxicological properties of ginger (Zingiber officinale Roscoe): a review of recent research	Food and Chemical Toxicology	2.4	0.8	500	55.6	5.6
48*	2001	Banskota A.H., Tezuka Y., Kadota S. Recent progress in pharmacological research of propolis	Phytotherapy Research	0.7	0.4	499	31.2	2.3
49	2005	Lindequist U., Niedermeyer T.H.J., Jülich W.-D. The pharmacological potential of mushrooms	Evidence-based Complementary and Alternative Medicine	0	0.0	491	40.9	9.2
50	2004	Li W.L., Zheng H.C., Bukuru J., De Kimpe N. Natural medicines used in the traditional Chinese medical system for therapy of diabetes mellitus	Journal of Ethnopharmacology	1.5	0.8	486	37.4	2.8
51	1988	Rios J.L., Recio M.C., Villar A. Screening methods for natural products with antimicrobial activity: a review of the literature	Journal of Ethnopharmacology	0.5	NA	482	16.6	NA
52	1998	Eloff J.N. Which extractant should be used for the screening and isolation of antimicrobial components from plants?	Journal of Ethnopharmacology	0.6	NA	481	25.3	2.4
53	2002	Roth B.L., Baner K., Westkaemper R., Siebert D., Rice K.C., Steinberg S., Ernsberger P., Rothman R.B. Salvinorin A: a potent naturally occurring nonnitrogenous κ opioid selective agonist	Proceedings of the National Academy of Sciences of the United States of America	10.7	7.2	478	31.9	3.6
54	2009	Wagner H., Ulrich-Merzenich G. Synergy research: approaching a new generation of phytopharmaceuticals	Phytomedicine	2.2	0.9	478	59.8	15.9
55	2004	Chattopadhyay I., Biswas K., Bandyopadhyay U., Banerjee R.K. Turmeric and curcumin: biological actions and medicinal applications	Current Science	0.7	0.3	469	36.1	1.3
56	2002	Biswas K., Chattopadhyay I., Banerjee R.K., Bandyopadhyay U. Biological activities and medicinal properties of neem (Azadirachta indica)	Current Science	0.6	0.3	465	31.0	1.5
57	1998	Ernst E. Harmless herbs? A review of the recent literature	American Journal of Medicine	4.5	NA	458	24.1	21.0
58	2002	Raskin I., Ribnicky D.M., Komarnytsky S., Ilic N., Poulev A., Borisjuk N., Brinker A., Moreno D.A., Ripoll C., Yakoby N., O'Neal J.M., Cornwell T., Pastor I., Fridlender B. Plants and human health in the twenty-first century	Trends in Biotechnology	6.3	1.7	456	30.4	9.8
59	2003	Gülçin I., Oktay M., Kireçci E., Küfrevioglu Ö.I. Screening of antioxidant and antimicrobial activities of anise (*Pimpinella anisum* L.) seed extracts	Food Chemistry	1.3	0.9	454	32.4	7.1
60	2002	Choi C.W., Kim S.C., Hwang S.S., Choi B.K., Ahn H.J., Lee M.Y., Park S.H., Kim S.K. Antioxidant activity and free radical scavenging capacity between Korean medicinal plants and flavonoids by assay-guided comparison	Plant Science	1.6	0.8	446	29.7	3.5
61	2000	Scartezzini P., Speroni E. Review on some plants of Indian traditional medicine with antioxidant activity	Journal of Ethnopharmacology	0.6	0.5	444	26.1	2.7
62*	2003	Chainani-Wu N. Safety and anti-inflammatory activity of curcumin: a component of tumeric (*Curcuma longa*)	Journal of Alternative and Complementary Medicine	1.0	0.4	443	31.6	14.2
63	1999	Gübitz G.M., Mittelbach M., Trabi M. Exploitation of the tropical oil seed plant *Jatropha curcas* L.	Bioresource Technology	0.9	0.7	442	24.6	2.6
64	2003	Strobel G.A. Endophytes as sources of bioactive products	Microbes and Infection	3.8	1.7	442	31.6	1.5
65	2008	Wang L., Zhou G.-B., Liu P., Song J.-H., Liang Y., Yan X.-J., Xu F., Wang B.-S., Mao J.-H., Shen Z.-X., Chen S.-J., Chen Z. Dissection of mechanisms of Chinese medicinal formula Realgar-indigo naturalis as an effective treatment for promyelocytic leukemia	Proceedings of the National Academy of Sciences of the United States of America	9.4	6.9	430	47.8	5.0
66*	2002	Kronenberg F., Fugh-Berman A. Complementary and alternative medicine for menopausal symptoms: a review of randomized, controlled trials	Annals of Internal Medicine	11.5	4.1	429	28.6	19.7
67	2000	Calixto J.B. Efficacy, safety, quality control, marketing and regulatory guidelines for herbal medicines (phytotherapeutic agents)	Brazilian Journal of Medical and Biological Research	0.7	0.4	429	25.2	1.7
68	2000	Stermitz F.R., Lorenz P., Tawara J.N., Zenewicz L.A., Lewis K. Synergy in a medicinal plant: antimicrobial action of berberine potentiated by 5'-methoxyhydnocarpin, a multidrug pump inhibitor	Proceedings of the National Academy of Sciences of the United States of America	10.8	7.8	425	25.0	3.8
69	2006	Pourmorad F., Hosseinimehr S.J., Shahabimajd N. Antioxidant activity, phenol and flavonoid contents of some selected Iranian medicinal plants	African Journal of Biotechnology	0	0.3	423	38.5	3.9
70	2005	Kanadaswami C., Lee L.-T., Lee P.-P.H., Hwang J.-J., Ke F.-C., Huang Y.-T., Lee M.-T. The antitumor activities of flavonoids	*In Vivo*	1.1	0.5	423	35.3	10.0
71	2007	Anwar F., Latif S., Ashraf M., Gilani A.H. Moringa oleifera: a food plant with multiple medicinal uses	Phytotherapy Research	1.5	0.6	417	41.7	2.5
72	2002	Sabu M.C., Kuttan R. Anti-diabetic activity of medicinal plants and its relationship with their antioxidant property	Journal of Ethnopharmacology	1.2	0.9	408	27.2	4.0
73*	2000	Mishra L.-C., Singh B.B., Dagenais S. Scientific basis for the therapeutic use of *Withania somnifera* (ashwagandha): a review	Alternative Medicine Review	0.0	0.3	408	24.0	3.1
74	2003	Javanmardi J., Stushnoff C., Locke E., Vivanco J.M. Antioxidant activity and total phenolic content of Iranian Ocimum accessions	Food Chemistry	1.3	0.9	405	28.9	5.0
75	2000	Moreno M.I.N., Isla M.I., Sampietro A.R., Vattuone M.A. Comparison of the free radical-scavenging activity of propolis from several regions of Argentina	Journal of Ethnopharmacology	0.6	0.5	403	23.7	1.6
76	2002	Lu Y., Yeap Foo L. Polyphenolics of Salvia—a review	Phytochemistry	1.7	1.0	402	26.8	2.0
77	2006	Cai Y.-Z., Mei Sun, Jie Xing, Luo Q., Corke H. Structure-radical scavenging activity relationships of phenolic compounds from traditional Chinese medicinal plants	Life Sciences	2.4	1.0	399	36.3	8.3
78*	1988	Grunberger D., Banerjee R., Eisinger K., Oltz E.M., Efros L., Caldwell M., Estevez V., Nakanishi K. Preferential cytotoxicity on tumor cells by caffeic acid phenethyl ester isolated from propolis	Experientia	1.2	NA	398	13.7	NA
79*	2004	O'Neill P.M., Posner G.H. A medicinal chemistry perspective on artemisinin and related endoperoxides	Journal of Medicinal Chemistry	5.1	2.0	397	30.5	8.6
80	2006	Aggarwal S., Ichikawa H., Takada Y., Sandur S.K., Shishodia S., Aggarwal B.B. Curcumin (diferuloylmethane) down-regulates expression of cell proliferation and antiapoptotic and metastatic gene products through suppression of IκBα kinase and Akt activation	Molecular Pharmacology	4.5	2.6	391	35.5	16.2
81	2006	Prabuseenivasan S., Jayakumar M., Ignacimuthu S. *In vitro* antibacterial activity of some plant essential oils	BMC Complementary and Alternative Medicine	0.0	0.5	390	35.5	10.1
82	2003	Pervaiz S. Resveratrol: From grapevines to mammalian biology	FASEB Journal	7.2	3.7	388	27.7	6.2
83*	1988	Sparnins V.L., Barany G., Wattenberg L.W. Effects of organosulfur compounds from garlic and onions on benzo[a]pyrene-induced neoplasia and glutathione s-transferase activity in the mouse	Carcinogenesis	2.6	NA	381	13.1	NA
84*	2004	van der Heijden R., Jacobs D.I., Snoeijer W., Hallard D., Verpoorte R. The Catharanthus alkaloids: pharmacognosy and biotechnology	Current Medicinal Chemistry	4.4	1.7	379	29.2	4.6
85	1998	Alarcon-Aguilara F.J., Roman-Ramos R., Perez-Gutierrez S., Aguilar-Contreras A., Contreras-Weber C.C., Flores-Saenz J.L. Study of the anti-hyperglycemic effect of plants used as antidiabetics	Journal of Ethnopharmacology	0.6	NA	379	19.9	1.5
86*	2005	Shishodia S., Sethi G., Aggarwal B.B. Curcumin: getting back to the roots	Annals of the New York Academy of Sciences	2.0	1.0	376	31.3	20.7
87	2004	Grover J.K., Yadav S.P. Pharmacological actions and potential uses of *Momordica charantia*: a review	Journal of Ethnopharmacology	1.5	0.8	375	28.8	2.4
88	1998	Chatterjee S.S., Bhattacharya S.K., Wonnemann M., Singer A., Müller W.E. Hyperforin as a possible antidepressant component of hypericum extracts	Life Sciences	2.0	NA	375	19.7	17.0
89	1997	Vaya J., Belinky P.A., Aviram M. Antioxidant constituents from licorice roots: isolation, structure elucidation and antioxidative capacity toward LDL oxidation	Free Radical Biology and Medicine	3.6	NA	373	18.7	4.0
90	2000	Liu F., Ng T.B. Antioxidative and free radical scavenging activities of selected medicinal herbs	Life Sciences	1.9	0.7	371	21.8	4.6
91	2002	Holetz F.B., Pessini G.L., Sanches N.R., Cortez D.A.G., Nakamura C.V., Dias Filho B.P. Screening of some plants used in the Brazilian folk medicine for the treatment of infectious diseases	Memorias do Instituto Oswaldo Cruz	0.7	0.5	369	24.6	2.6
92	1998	Heinrich M., Ankli A., Frei B., Weimann C., Sticher O. Medicinal plants in Mexico: healers' consensus and cultural importance	Social Science and Medicine	0.0	NA	367	19.3	3.1
93*	2004	Smit A.J. Medicinal and pharmaceutical uses of seaweed natural products: a review	Journal of Applied Phycology	0.8	0.5	367	28.2	2.2
94	2006	Li Y., Guo C., Yang J., Wei J., Xu J., Cheng S. Evaluation of antioxidant properties of pomegranate peel extract in comparison with pomegranate pulp extract	Food Chemistry	2.5	1.4	366	33.3	7.4
95*	2003	Normile D. The new face of traditional Chinese medicine	Science	29.8	11.2	361	25.8	7.5
96	2005	Salem M.L. Immunomodulatory and therapeutic properties of the Nigella sativa L. seed	International Immunopharmacology	2.1	0.8	360	30.0	1.7
97	2001	Araújo C.A.C., Leon L.L. Biological activities of *Curcuma longa* L	Memorias do Instituto Oswaldo Cruz	0.7	0.4	358	22.4	3.1
98	2001	Srinivasan D., Nathan S., Suresh T., Lakshmana Perumalsamy P. Antimicrobial activity of certain Indian medicinal plants used in folkloric medicine	Journal of Ethnopharmacology	0.8	0.7	356	22.3	1.7
99	2009	López-Lázaro M. Distribution and biological activities of the flavonoid luteolin	Mini-Reviews in Medicinal Chemistry	3.0	1.0	354	44.3	11.4
100	2009	Ravindran J., Prasad S., Aggarwal B.B. Curcumin and cancer cells: How many ways can curry kill tumor cells selectively?	AAPS Journal	3.6	1.4	353	44.1	8.0

NA, not available. Impact factor and SJR were firstly available in 1975 and 1999, respectively. Hence, there are articles with impact factor or SJR labeled as “NA.” An asterisk “*” indicates that the article was not freely available to the authors through various schemes at the time of the literature analysis (November 2017).

The 100 articles were published in 59 journals with impact factors ranging from 0 to 29.8 (mean ± SD: 2.7 ± 3.8). Half of the 100 articles were published either in a journal dedicated to ethnopharmacology and medicinal plant research (*n* = 28) or a clinical journal (*n* = 19; Table 2). Only one article was published in a social sciences journal. Four core journals published 33 articles; 21 and 34 journals, respectively, published the next 33 and the last 33 articles (4:21:34). This indicated that the distribution of publications did not fulfill Bradford's law. The four core journals were *Journal of Ethnopharmacology* (*n* = 17), *Food Chemistry* (*n* = 7), *Life Sciences* (*n* = 5), and *Journal of Agricultural and Food Chemistry* (*n* = 4).

**Table 2:** Journals in which the 100 most-cited ethnopharmacology articles were published.

**Journal**	**Publication count**	**Citation count**	**Citation per article**	**Topic**
Journal of Ethnopharmacology	17	9,198	541	2
Food Chemistry	7	3,873	553	1
Life Sciences	5	3,010	602	2
Journal of Agricultural Food Chemistry	4	5,973	1,493	1
Phytochemistry	3	1,612	537	3
Phytotherapy Research	3	1,421	474	2
Proceedings of the National Academy of Sciences of the United States of America	3	1,333	444	6
Science	2	1,984	992	6
African Journal of Biotechnology	2	1,189	595	7
Current Medicinal Chemistry	2	1,183	592	3
Diabetes Care	2	1,067	534	5
Current Science	2	934	467	6
Memorias do Instituto Oswaldo Cruz	2	727	364	4
Journal of the American Medical Association	1	5,253		5
Journal of Applied Microbiology	1	1,232		7
Applied Microbiology and Biotechnology	1	1,162		7
Phytochemical Analysis	1	879		3
Journal of Association of Physicians of India	1	819		5
Journal of Natural Products	1	797		3
Angewandte Chemie International Edition	1	770		3
Drug Discovery Today	1	736		3
Environmental Health Perspectives	1	720		4
Bulletin of the World Health Organization	1	705		5
Toxicon	1	689		4
Advances in Experimental Medicine and Biology	1	669		4
Molecular Aspects of Medicine	1	651		4
Brazilian Journal of Microbiology	1	591		7
Pharmacognosy Reviews	1	586		2
European Journal of Cancer	1	566		4
Critical Reviews in Immunology	1	541		4
PLoS ONE	1	530		6
Nature	1	519		6
Mutation Research—Fundamental and Molecular Mechanisms of Mutagenesis	1	507		4
Food and Chemical Toxicology	1	500		4
Evidence-Based Complementary and Alternative Medicine	1	491		2
Phytomedicine	1	478		2
American Journal of Medicine	1	458		5
Trends in Biotechnology	1	456		7
Plant Science	1	446		8
Journal of Alternative and Complementary Medicine	1	443		2
Bioresource Technology	1	442		7
Microbes and Infection	1	442		7
Annals of Internal Medicine	1	429		5
Brazilian Journal of Medical and Biological Research	1	429		5
*In Vivo*	1	423		5
Alternative Medicine Review	1	408		5
Experientia	1	398		5
Journal of Medicinal Chemistry	1	397		3
Molecular Pharmacology	1	391		5
BMC Complementary and Alternative Medicine	1	390		2
FASEB Journal	1	388		5
Carcinogenesis	1	381		5
Annals of the New York Academy of Sciences	1	376		6
Free Radical Biology and Medicine	1	373		5
Journal of Applied Phycology	1	367		8
Social Science and Medicine	1	367		9
International Immunopharmacology	1	360		4
Mini-Reviews in Medicinal Chemistry	1	354		3
AAPS Journal	1	353		5

*The topics of the journals were coded as 1, food research (chemistry etc.)(number of articles, n = 11); 2, ethnopharmacology and medicinal plant research (n = 28); 3, phytochemistry with a link to traditional uses (n = 11); 4, pharmacology and pharmacy (n = 10); 5, clinical (n = 20); 6, multidisciplinary journals (n = 10); 7, microbiology and biotechnology (n = 7); 8, plant science (n = 2); and 9, social sciences (n = 1)*.

### What key research themes were relevant (as indicated by citations) in ethnopharmacology?

**Figure 1:**
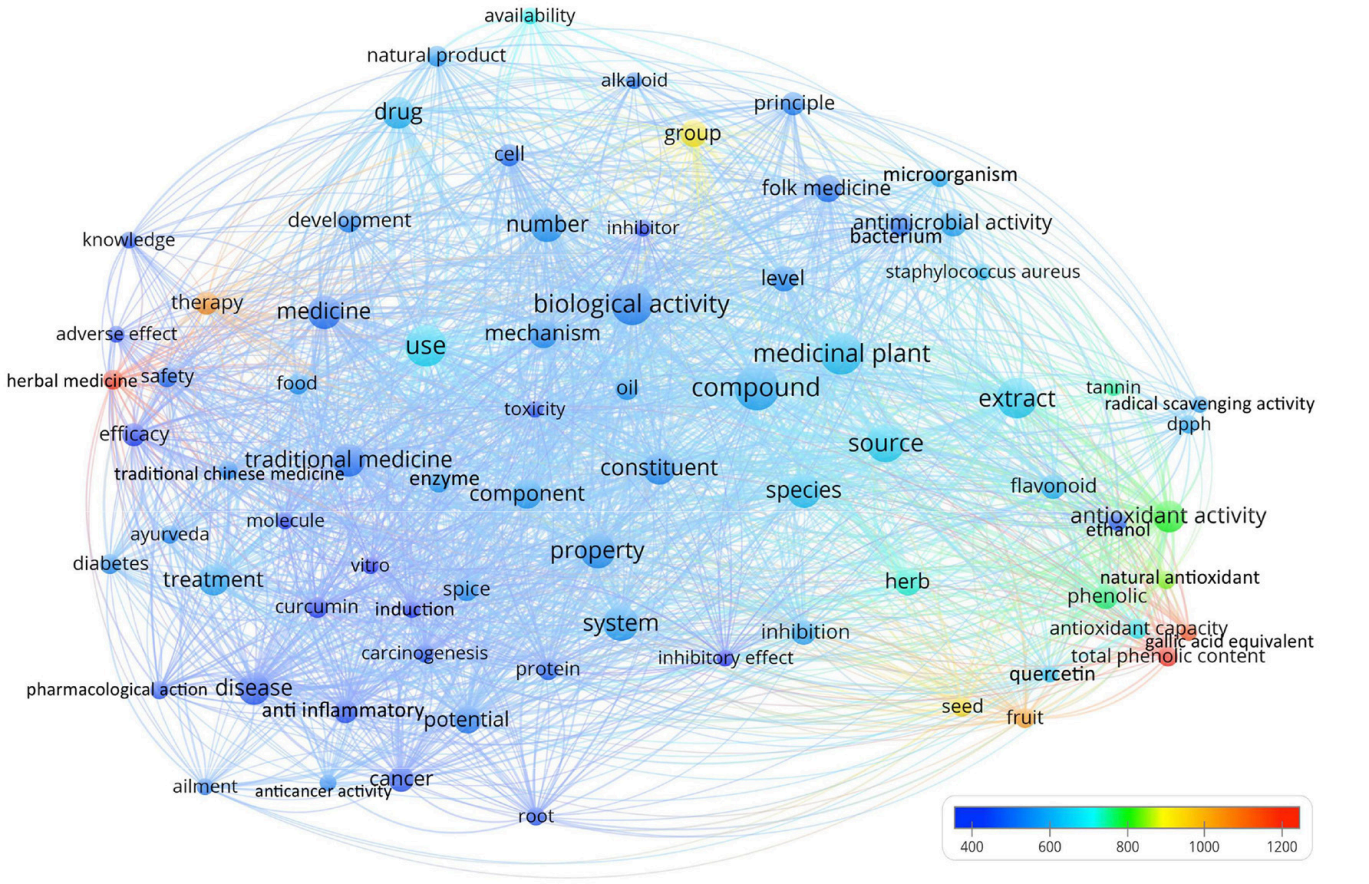
Term map using words from titles and abstracts of the 100 most-cited ethnopharmacology articles. Words from titles and abstracts were parsed, analyzed and visualized by VOSviewer. There were 76 terms that appeared in five or more articles and hence included in the term map. Each bubble represents a term or phrase. The bubble size indicates its frequency of occurrence. The bubble color indicates the averaged citation counts received by articles containing the term or phrase. A line connects two bubbles if they co-occurred in any of the 100 articles. If two terms co-occurred more frequently, the two bubbles would be in closer proximity. Irrelevant terms were removed manually upon visual inspection of the initial map generated. Lower right corner contains numerous terms and phrases related to anti-oxidant activity and articles containing them had more citations than the average, as indicated by the red, orange, yellow, and green bubbles.

There were 76 terms or phrases that appeared in five or more of the 100 articles (Figure 1). Figure 1 clearly suggested that terms or phrases related to anti-oxidant effects received much more citations per article than the average, as reflected by the red-orange-yellow-green bubbles concentrated on the lower right corner of the term map. For instance, “total phenolic content” appeared in seven articles with an average of 1,175 citations each. “Gallic acid equivalent” appeared in five articles with an average of 1,126 citations each. “Antioxidant activity” appeared in 18 articles with an average of 797 citations each. “DPPH (2,2-diphenyl-1-picrylhydrazyl)” appeared in six articles with an average of 600 citations each. Free radical scavenging activity appeared in five articles with an average of 592 citations each. Meanwhile, seven articles had the phrase “herbal medicine” with an average of 1,148 citations each. Certain terms may reflect the geographical content of the ethnopharmacology articles, such as “traditional Chinese medicine” (*n* = 6) relates to China and “Ayurveda” (*n* = 6) relates to India. The 20 terms that appeared most in the titles and abstracts of the 100 articles are listed in Table 3.

**Table 3:** The 20 terms that appeared most in the titles and abstracts of the 100 articles.

**Terms**	**Appearance**	**Citations per article**
Compound	35	604
Medicinal plant	32	640
Biological activity	31	547
Use	31	679
Extract	29	658
Source	26	663
Property	22	567
System	22	588
Number	21	573
Medicine	20	522
Species	20	640
Traditional medicine	19	520
Antioxidant activity	17	797
Constituent	18	558
Disease	18	482
Drug	18	622
Treatment	17	596
Component	16	588
Mechanism	15	566
Antimicrobial activity	13	596

Studies with a focus on antioxidant effects, and works focusing on fruits and other food-related species and their related concepts were often highly cited. This may be linked to the fact that antioxidant tests are usually affordable and easily available (Kedare and Singh, 2011) so the majority of the labs, regardless of their budget size, could readily use them. It also reflects the size of research fields—the food sciences field is much larger than research into medicinal plants (as well as the associated industries). The food sitting close to traditional medicine makes an interesting link to the “Let food be thy medicine and medicine be thy food” concept advocated by Hippocrates. Functional foods have an enormous global market that has recently grown from US$33 billion (Menrad, 2003) to US$168 billion (Vicentini et al., 2016). A substantive body of research on functional food research advocates the intake of antioxidant nutrients for better well-being and health (Diplock et al., 1998). Food plants are also easily and often commercially available through the world. In other words, resource availability may be the key concept to interpret the focus for antioxidants and food plants in general. Spices are an important topic in these studies, particularly the curcumin from turmeric (*Curcuma longa* L.), which was mentioned in eight of the 100 articles, including in the titles of six of the 100 articles. This is not unexpected, since many spices are heavily used in the traditional medicine (Vimala et al., 1999; Aggarwal et al., 2007; Singh, 2007). While this demonstrates the importance of the topic in bibliometric terms, readers should be aware that antioxidant tests are often based on simple chemical reactions that may not translate well to demonstrate effects in real biological systems. Currently, they are not accepted as a relevant aspect of pharmacological research (see e.g., the “Rules of Five” of the *Journal of Ethnopharmacology*)^4^.

The 100 articles involved clinical or pre-clinical research on numerous diseases. Diseases often mentioned in the 100 articles included diabetes (*n* = 7; 565 citations each) and cancer (*n* = 12; 473 citations each). The citations of these papers are mainly from the year 2004 onwards. For instance, there is a comprehensive report that reviewed the effects of various medicinal plants originating from Asia, Europe, the Middle East, and the Americas on diabetes (Bailey and Day, 1989). Similarly, there was a report on plant-derived compounds as anti-cancer agents (Cragg and Newman, 2005).

Other diseases that were mentioned in at least three of the 100 articles included Alzheimer's disease (*n* = 3; 564 citations each), arthritis (*n* = 4; 478 citations each), and rheumatism (*n* = 3; 449 citations each). In addition, six papers mentioned gastrointestinal disease (three of them were in the abstracts while the other three were in keywords). One paper mentioned respiratory disease. Since these terms appeared in fewer than five of the 100 articles' titles or abstracts, they were not visualized in Figure 1. No paper mentioned gynecology or dermatology. Some studies show some links into traditional medicine/ethnopharmacology, but are in essence a clinical study, for example (Kronenberg and Fugh-Berman, 2002).

Some important metabolites and related concepts have been mentioned at least thrice in the 100 articles, including alkaloids (*n* = 5; on average 534 citations), essential oil (*n* = 4; 774), flavonoids (*n* = 11; 621), and tannin (*n* = 5; 752). These topics have constituted a large portion of the existing ethnopharmacology literature.

The link of the 100 ethnopharmacological papers into the context of drug discovery seemed to be marginal. Seven of the 100 papers discussed the concept of drug discovery in their titles, abstracts, or keywords. Six of the seven papers reviewed the drug discovery from Allium (garlic and onion), seaweed, medicinal plants, and natural products in general (Block, 1992; Fabricant and Farnsworth, 2001; Smit, 2004; Balunas and Kinghorn, 2005; Gurib-Fakim, 2006; Harvey, 2008). Klayman (1985) analyzed the development of artemisinin from the Chinese medicinal plant *Artemisia annua* L. The remaining paper demonstrated a possible major active metabolite responsible for the antidepressant effect of the extract from St. John's wort (Chatterjee et al., 1998), which happens to be the only paper dealing with St. John's wort, one of the best studied medicinal plants. Together with a paper dealing with opioid K inhibition and salvinorin (Roth et al., 2002), these two are the only ones dealing with neuropharmacology among the 100. Another interesting gap is research on Cannabis and its active metabolites tetrahydrocannabinol (THC) and cannabis (CBD). Here a large number of papers have been published, but clearly not deemed to be linked to ethnopharmacology, for example (Bisogno et al., 2001). Sustainability, access, and benefit sharing are also clearly are not major topics (as evident from Figure 1, the only term with relevance in this context seems to be “availability”).

The latest papers in our top 100 list were published in 2010. Our Scopus search has identified that only two post-2010 papers had at least 300 citations, and both of them have exceeded the citation count of the last paper on our top 100 list. However, they were excluded as one focused on genomics and drug synthesis (Cragg and Newman, 2013) (582 citations) whereas the other one strictly dealt with chemistry (Kumar and Pandey, 2013; 399 citations).

The citation counts of the articles have no significant correlation with the number of years since publication (*r* = 0.134, *p* = 0.184). Since the articles were generally published well before 2010, an analysis of open vs. closed access is problematic and was not conducted. Eight articles became available under an open access (OA) model at the time of publication, but today 66 more articles are available freely through different schemes. There seems to be no major difference in the number of OA articles among the highly cited and the less cited (Top 100: 8 OA articles, Rank 1000–1100: 9 OA articles, Rank 1900–2000: 5 OA articles). This is in line with the findings from life science research that open access might be associated with more full text downloads without an actual citation advantage (Davis et al., 2008).

### What can we learn from the highly cited papers in this field in the context of ethnopharmacology's wider and changing relevance within the natural sciences?

The 100 articles were binarized into pre-2000 (the year 2000 inclusive) and post-2000 publications and assessed. There were 32 pre-2000 and 68 post-2000 publications. The topics of pre-2000 publications appeared to be more focused, with only several specific plants or food appearing in multiple (i.e., two or more) articles, namely garlic, onion, propolis, rosemary, sage and *Withania somnifera* (L.) Dunal. Each appeared in two articles published pre-2000. The foci of post-2000 publications, on the other hand, seemed to be more diversified and involved more plants or food, namely aloe vera, cinnamon, clove, *Coccinia grandis* (L.) Voigt, *C. longa* L., Eucalyptus, ginger, *Gymnema sylvestre* (Retz.) R.Br. ex Sm., *Momordica charantia* L., mushroom, origanum, pomegranate, sage (*Salvia officinalis* L.), thyme and yeast. Except for *C. longa, M. charantia* and yeast that have had three appearances each, the other species and foods have appeared twice in the post-2000 publications. The post-2000 publications have shifted foci compared to their pre-2000 counterparts, with the evidence that sage is the only plant that has appeared multiple times both in pre-2000 and post-2000 publications.

Another noticeable point is that post-2000 publications had evaluated wider aspects of effects of the medicinal plants and functional food. For instance, post-2000 publications focus more on antibacterial, anticancer, anticandidal, antifungal, antimicrobial, antioxidant, and antitumor activities, while pre-2000 publications only referred to antibacterial, antimicrobial, and antioxidant activities.

### Who and what has driven the research?

The 100 most-cited articles were contributed by a total of 159 authors. Only one author, Bharat Bhushan Aggarwal, has contributed to four publications making him the only one to contribute three or more of the 100 most-cited articles. He authored four of them. All of them were published post-2000. No author has multiple contributions to the pre-2000 articles. The 100 articles were contributed by 160 affiliations. Those with three or more articles were University of Texas M. D. Anderson Cancer Center (*n* = 5), The University of Hong Kong (*n* = 3), and University of Illinois at Chicago (*n* = 3). All but one of these 11 articles were published post-2000. No affiliation has multiple contributions to the pre-2000 articles.

The 100 articles originated from 40 countries/territories, with the top contributions being by The United States (*n* = 29) and India (*n* = 13) (Table 4). Also noticeable countries/territories were China (*n* = 8), the United Kingdom (*n* = 8), Brazil (*n* = 5), and Germany (*n* = 5). In summary, the 100 articles had major contributions, not mutually exclusive, from countries/territories from Europe (*n* = 41), Asia (*n* = 39), and North America (*n* = 32), and some contributions from South America (*n* = 6), Africa (*n* = 6), and Oceania (*n* = 2). Comparing the articles published pre- and post-2000, The United States, India, and United Kingdom were listed among the top 5 contributors in both periods. However, China and Iran have a much larger contribution in the post-2000 publications (China: *n* = 7, Iran: *n* = 4) compared to the pre-2000 publications (China: *n* = 1, Iran: *n* = 0), and they have consequently replaced Brazil and Germany as members of the top five contributors.

**Table 4:** Countries/territories contributed to the 100 most cited ethnopharmacology articles.

**Country/territory**	**Number of articles**
United States	29
India	13
China	8
United Kingdom	8
Brazil	5
Germany	5
Hong Kong SAR (China)	4
Iran	4
Israel	4
Netherlands	3
South Africa	3
Spain	3
Turkey	3
Belgium	2
Canada	2
Japan	2
Poland	2
Singapore	2
South Korea	2
Ukraine	2

*Countries/territories that each contributed to one article included Algeria, Argentina, Australia, Austria, Bulgaria, Croatia, Denmark, Finland, France, Italy, Lithuania, Mauritius, Mexico, New Zealand, Nigeria, Oman, Pakistan, Switzerland, Taiwan, and United Arab Emirates*.

Geography and culture have been an integral part of ethnopharmacology. Interestingly, only one of the 100 articles related to the social sciences aspects, which examined the use of medicinal plants in four indigenous groups of Mexican Indians (Heinrich et al., 1998). Perhaps in the future, more attention should be directed toward the cultural aspects of ethnopharmacology.

### Limitations

The list of top 100 most cited articles, by its nature, has inherent limitations. The first and foremost limitation is the search strategy. An article would not be identified if its title, abstract, and keywords did not contain the pre-defined search terms. The second limitation is the definition of ethnopharmacology. For instance, one paper (Chatterjee et al., 1998) reported on the efficacy of the extract from a licensed European medicinal plant (St. John's wort) as antidepressant, and it surely deals with neuropharmacology, but it may or may not constitute ethnopharmacology *sensu stricto*. Meanwhile, papers would be excluded from the current study if they were classed strictly as drug discovery or the title, abstracts or keywords assigned by EMTREE or MeSH did not contain the pre-defined key words related to ethnopharmacology. For example, some very widely used reviews on drug discovery from natural sources are not coded in such a way that they are included in this list: (Newman et al., 2003; Newman and Cragg, 2007) (These papers have 1,860 and 2,594 citations recorded by Scopus, respectively). This suggests a need for a top-level keyword (such as ethnopharmacology or traditional medicine) to be used widely. Ethnopharmacology or, more generally, the information which forms the basis of research and development activities, is still not well-recognized as an important element of the industrial development pipeline. This contradicts the common perception that ethnopharmacology is seen as an important source for new medicines. In essence, this also reflects the classical separation of the natural sciences into defined disciplines like chemistry and pharmacology, with ethnopharmacology being more strongly linked to pharmacology (and food science). Lastly, this work is based on a retrospective analysis and highlights research interests of the last decade or the last two decades and will be changing citation preferences in the future. It can be argued that the field may be shifting away from antioxidants and food science as some journals are now rejecting manuscripts based solely on *in silico* or simple antioxidant assays of food (Harnly, 2017). This also includes Frontiers in Pharmacology (section Ethnopharmacology) and the Journal of Ethnopharmacology. However, this latest shift has yet to be reflected in terms of citations.

### What can we learn from this for the future development of the field?

The research into bioactive natural products from medicinal plants has a long tradition, with many groups in the nineteenth century starting to search for the active principles in medicinal products and with an interest in the species pharmacology (Heinrich and Jäger, 2015). With the booming literature in neuroscience and nutritional neuroscience (Yeung et al., 2017b), it would not be surprising to see stronger links of ethnopharmacology to neuroscience, neuropharmacology and food science in the near future. This would also bring ethnopharmacology back to its “roots,” since the early developments were driven by scientific interests in psychoactive plants (Heinrich, 2013). For instance, future research may shed light on how ingredients from medicinal plants would modulate, or even treat, eating or neuropsychiatric disorders. Such effects may be manifested as brain perfusion changes recorded by functional magnetic resonance imaging (Yeung et al., 2016, 2018). Metabolic syndrome and diabetes will continue to be a key area of interest and will strengthen the links into preventive medicine and food sciences. In future, this field should move away further from papers focused solely on *in silico* or on simple antioxidant screening reports to papers introducing new methods applicable to ethnopharmacology, which is largely missing from the current top 100 list.

## Conclusions

We have identified the 100 most-cited articles in ethnopharmacology. This analysis identifies what has been seen to be of such importance in scientific terms that it is commonly used in citations. Some surprising outcomes include the important link to food sciences, and the relevance of some biological assays, which, in reality, are not considered to be of pharmacological relevance. Some other areas are notably absent including stronger links into neuropharmacology and a lack appreciation of social and cultural sciences approaches. The analysis demonstrated that ethnopharmacology is at the crossroads of several disciplines (most notably pharmacology and food science) and it is the ethnopharmacologists' challenge and opportunity to define this area in new ways, including the development of new links and foci (cf. Nina Etkin's work, e.g., Etkin and Ross, 1991). In a book published in 2015 a large number of contributors provided a short definition of what they consider ethnopharmacology to be Heinrich and Jäger (2015). The diversity of their responses with foci on pharmacology, clinical research, cultural sciences and studies, biological sciences and environmental research are reflected in the diversity seen in this bibliometric analysis. This diversity clearly is one of the key strengths, but also a challenge of ethnopharmacology, which sees itself to be inter- or trans-disciplinary.

## Author contributions

AY, MH, and AA: conceived the work; AY: acquired data and drafted the work; AY, MH, and AA: analyzed data; MH and AA: critically revised the work. All authors have approved the final content of the manuscript.

### Conflict of interest statement

The authors declare that the research was conducted in the absence of any commercial or financial relationships that could be construed as a potential conflict of interest.

## Footnotes

^1^https://www.journals.elsevier.com/journal-of-ethnopharmacology/

^2^https://www.frontiersin.org/journals/pharmacology/sections/ethnopharmacology#about

^3^FWCI shows how well cited an article is as compared to similar ones. It considers the publication year, article type and disciplines associated with its source. It is the ratio of the total citations actually received by the article to the total citations that would be expected based on the average of the subject field. For instance, a FWCI of 1.50 means the article was cited 50% more times than expected.

^4^https://www.sciencedirect.com/science/article/pii/S0378874105008251
